# The Role of Subcutaneous Furosemide in Heart Failure Management: A Systematic Review

**DOI:** 10.1007/s11886-024-02124-4

**Published:** 2024-10-01

**Authors:** Wynne Widiarti, Pandit Bagus Tri Saputra, Melissa Valentina Ariyanto, Cornelia Ghea Savitri, Chaq El Chaq Zamzam Multazam, Johanes Nugroho Eko Putranto, Firas Farisi Alkaff

**Affiliations:** 1 Faculty of Medicine, Universitas Airlangga, Surabaya, Indonesia; 2 Department of Cardiology and Vascular Medicine, Faculty of Medicine, Universitas Airlangga - Dr.Soetomo General Academic Hospital, Surabaya, Indonesia; 3 Cardiovascular Research and Innovation Center, Universitas Airlangga, Surabaya, Indonesia; 4 National Heart and Lung Institute, Imperial College London, London, UK; 5 Division of Nephrology, Department of Internal Medicine, University of Groningen, University Medical Center Groningen, Groningen, Netherlands; 6 Division of Pharmacology and Therapy, Department of Anatomy, Histology, and Pharmacology, Faculty of Medicine, Universitas Airlangga, Surabaya, Indonesia

**Keywords:** Heart failure, Acute heart failure, Subcutaneous furosemide, Subcutaneous diuretic

## Abstract

**Background:**

Acute decompensated heart failure (ADHF) patients with symptomatic congestion often require in-hospital admission for intravenous (IV) diuretic, impacting both patient well-being and healthcare expenses. Subcutaneous (SC) furosemide has a potential to facilitate outpatient management of ADHF patients. Thus, this study aims to assess the efficacy and safety of SC furosemide utilization, offering a potential alternative to traditional IV administration.

**Methods:**

A systematic search was conducted until April 14 2024 across scientific databases. This review included studies comparing SC furosemide with oral and IV formulations in adult HF patients.

**Results:**

This study analyzed 687 patients from 20 studies. The results demonstrate that SC furosemide can effectively manage symptomatic congestion in HF patients and results in significant cost reductions, symptom relief, and improved quality of life. Although further investigation into mortality rates is needed, SC furosemide demonstrates efficacy comparable to IV furosemide in diuresis and weight loss, with similar bioavailability and natriuretic effects. Adverse events are generally minor, predominantly related to skin irritation. Innovative strategies, such as developing isotonic alkaline solutions and improved infusion devices, are being explored to address these challenges.

**Conclusion:**

SC furosemide offers a promising alternative for managing ADHF, particularly in symptomatic HF patients with volume overload. The integration of SC furosemide into routine clinical practice and future guidelines, could optimize the management of HF, reducing hospital admission and improving patient outcomes.

**Supplementary Information:**

The online version contains supplementary material available at 10.1007/s11886-024-02124-4.

## Background

Heart failure (HF) is a clinical syndrome of cardiac abnormalities, accompanied with elevated biomarkers or objective evidence of congestion. In 2017, HF was determined as a global pandemic, affecting 64.3 million individuals worldwide [1]. As the disease progresses, there is typically a rise in hospital admissions, affecting both patient well-being and healthcare expenses. Acute decompensated HF (ADHF) patients primarily seek medical help due to symptomatic congestion that requires diuretic treatments. The preferred treatment approach involves the utilization of intravenous (IV) diuretics, due to the inconsistent absorption of oral medications particularly diuretics. Interestingly, nearly half of admitted ADHF patients only require three days or less of in-hospital diuresis, indicating a potential alternative through outpatient management [2].

Furosemide is a commonly prescribed loop diuretic, used to treat electrolyte and volume disturbances. Oral administration is preferred for its ease, minimal discomfort, simplicity, better adherence, and lower cost. However, for patients with decreased consciousness, severe nausea and vomiting, required for rapid action, or impaired gastrointestinal absorption. In such cases, intravenous, intramuscular, rectal, or subcutaneous administration may be considered. The subcutaneous (SC) administration of furosemide has recently gained attention as an alternative when oral or intravenous routes are not desirable or possible. Until recently, the United States (US) the Food and Drug Administration (FDA) approved the use of subcutaneous (SC) administration of furosemide. SC furosemide was first employed in 2004, primarily for palliative care. It proved to be beneficial in symptom alleviation and decreasing hospital admission rates, particularly among individuals with advanced heart failure [3]. SC furosemide offers several advantages that allow patients to remain at home and improve their quality of life while continuing parenteral therapy and holds potential to shift decongestive therapy in HF patients. This review aims to evaluate the role of SC furosemide in HF management, providing novel evidence in guiding future advancements for optimal HF management.

## Methods

This study was conducted following the Preferred Reporting Items for Systematic Reviews and Meta-Analyses (PRISMA) guidelines 2020 (Supplementary 1) [4]. The review protocol has been registered in the International Prospective Register of Systematic Reviews (PROSPERO) (CRD42024535598).

## Eligibility Criteria

Considering the aim of this review is to summarize and synthesize the latest evidence regarding SCF, we applied flexible inclusion criteria and included all study types except for case reports and case series due to their lack of generalizability, absence of control groups, and insufficient methodological rigor. Screening for eligible studies was guided by the following inclusion criteria: [1] Any spectrum of HF patients aged ≥ 18 years; [2] Studies comparing SC furosemide to oral or intravenous furosemide and placebo; and [3] Studies reported in English. There were no restrictions on publication year. We excluded studies lacking accessible full text and those involving non-human subjects.

## Search Strategy and Selection of Studies

A systematic search was conducted until April 14, 2024, in scientific databases including PubMed, ScienceDirect, Web of Science, Springer, Cochrane, Clinicaltrials.gov, and Proquest. The utilized search terms were (“heart failure”) AND (“Subcutaneous Furosemide” OR “SCF”). Any duplicate results were resolved prior to reviewing the titles and abstracts. Subsequently, the retrieved articles were screened based on their titles and abstracts to determine their relevance according to predefined criteria. Two reviewers conducted the screening process independently In addition, additional relevant articles were identified through citation and gray literature searching.

## Article Extraction

Data extraction was conducted independently by two reviewers. If any data is unavailable, the respective authors of the related study will be contacted to obtain the necessary data and information. Data that were extracted was summarized in Table 1 and Supplementary Table 3. Any discrepancies were resolved by the third author.

**Table 1:** Baseline Characteristics of Included Studies

Author, Year	Study design	Study location	Sample (n)	Male n (%)	Age	BMI	Type of HF	NYHA Class	EF	SCr	eGFR	NT Pro BNP
Austin et al. 2013 [10]	Prospective cohort	United Kingdom	IV: 14 (56%) SC: 11 (44%)	IV: 12 (86%) SC: 4 (45%)	74.1 IV: 70.2 (44–88) SC: 80.9 (71–90)	IV BMI < 19: 1 (7%); BMI 19–23: 2 (14%); BMI > 23: 11 (79%)	N/A	NYHA III: 3 (23%); NYHA IIIb: 6 (46%); NYHA IV: 4 (31%)	N/A	N/A	N/A	Mean decrease of 12.5%
Bensimhon et al. 2023 [6]	Case control	United States	- Furoscix: 24 - Comparator: 66	57 (63.3%) - Furoscix: 15 (62.5%) - Comparator: 42 (63.6)	56.0 (18%) - Furoscix: 56.0 (20%) - Comparator: 57.5 (13%)	Furoscix: 44.4 (13.7)	- **Furoscix:** Systolic HF: 11 (45.8%) Diastolic HF: 10 (41.7%) - **Comparator** Systolic HF: 28 (42.4%) Diastolic HF: 29 (43.9%)	N/A	N/A	Furoscix: 1.35 ± 0.40	N/A	Furoscix: 823.4 ± 1043.9
Bensimhon et al. 2024 [7]	Case control	United States	- AT HOME-HF: 34 - FREEDOM-HF: 24	N/A	N/A	N/A	N/A	N/A	N/A	Increase in SCr was 0.11 ± 0.2	N/A	N/A
Birch et al. 2023 [11]	Retrospective cohort	United Kingdom	116	86 (66.2%)	79 ± 10	N/A	N/A	NYHA Class III: 36 (31%) NYHA Class IV: 80 (69%)	N/A	N/A	N/A	N/A
Brown et al. 2019 [12]	Cohort	United Kingdom	16	N/A	N/A	N/A	- Left ventricular systolic dysfunction (LVSD): 14 (87%)	N/A	N/A	N/A	N/A	N/A
Civera et al. 2022 [13]	Cohort	Spain	55	32 (58.2%)	79.0 ± 8.0	N/A	N/A	NYHA Class II: 2 (3.6%); NYHA Class III: 53 (96.4%)	48 (30–58)	1.5 ± 0.6	44.9 (29.2 – 60.2)	5218 (2856–10,878)
De boer et al. 2016 [14]	Prospective cohort	N/A	10	8 (80%)	69.9	N/A	N/A	N/A	N/A	N/A	N/A	1638
Felker et al. 2019 [15]	Prospective cohort	United Kingdom	- Inpatient (IP): 20 - Outpatient (OP): 20	N/A	- IP: 70 - OP: 61	N/A	N/A	N/A	-IP: 39 -OP: 35	1.4	N/A	N/A
Galindo-ocana et al. 2012 [9]	Retrospective cohort	Spain	- SC: 17 - IV: 27	- SC: 6 (35.3%) - IV: 11 (41.3%)	- SC: 84 (76–88.5) - IV: 83 (77–88)	N/A	N/A	N/A	N/A	N/A	N/A	N/A
Gilotra et al. 2018 [8]	Prospective cohort	United States	Total: 40 IV: 19 SQ: 21	- IV: 10 (53%) - SQ: 8 (28%)	- IV: 54 ± 13 - SQ: 59 ± 13	- IV: 39.7 ± 11.2 - SQ: 37.8 ± 11.3	N/A	**IV** NYHA Class II: 7 (37%) NYHA Class III: 11 (58%) NYHA Class IV: 1 (5%) **SQ** NYHA Class II: 5 (24%) NYHA Class III: 13 (62%) NYHA Class IV: 3 (14%)	- IV: 20 (20–55) - SQ: 25 (15–55)	- IV: 1.2 (0.9–1.6) - SQ: 1.3 (0.9–1.7)	- IV: 67 ± 32 - SQ: 57 ± 21	- IV: 1556 (198–3449) - SQ: 1545 (501–3123)
López‐Vilella et al. 2021 [16]	Prospective cohort	Spain	- SC: 10 - Oral: 17	- SC: 8 (80%) - Oral: 6 (35.3%)	- SC: 73 ± 9 - Oral: 72 ± 8	N/A	N/A	** SC** NYHA Class III-IV: 4 (40%) NYHA Class IV: 6 (60%) **Oral** NYHA Class III-IV: 11 (64.3%) NYHA Class IV: 6 (35.7%)	- SC: 5 (50%) - Oral: 7 (41.2%)	**SC** Pre: 2 ± 0.9 Post: 2.3 ± 1.5 **Oral** Pre: 1.7 ± 0.8 Post: 1.9 ± 0.7	N/A	**SC** Pre: 6925 ± 8186 Post: 9377 ± 9168 **Oral** Pre: 7536 ± 10,548 Post: 8094 ± 10,530
Lozano Bahamonde et al. 2018 [17]	Retrospective cohort	Spain	12	9 (75%)	80.25 ± 8.75	N/A	N/A	N/A	37.08 ± 10.90	N/A	26.75 ± 14.07	N/A
Mohr et al. 2018 [18]	Prospective Cohort	Canada	16	15 (93.8%)	68	N/A	N/A	NYHA Class I: 13 (81%)	N/A	N/A	N/A	N/A
Ojeifo et al. 2016 [19]	Prospective Cohort	United States	- Total: 12 - SC: 7 - IV: 5	- SC: 2 (71%) - IV: 4 (80%)	- SC: 60.3 - IV: 57.2	N/A	N/A	N/A	- SC: 4 (55%) - IV: 4 (80%)	N/A	N/A	N/A
Osmanska et al. 2024 [20]	Prospective Cohort	United Kingdom	- Bolus: 18 - Patch infusor:: 20	- Bolus: 13 (72%) - Patch infusor: 11 (55%)	- Bolus: 71 (64–74) - Patch infusor: 75 (64–85)	- Bolus: 32 (28–36) - Patch infusor: 30 (26–35)	N/A	**Bolus** - NYHA Class II: 16 (89%) - NYHA Class III: 2 (11%) - NYHA Class IV: 0 (0%) **Patch infusor** - NYHA Class II: 5 (25%) - NYHA Class III: 14 (70%) - NYHA Class IV: 1 (5%)	- Bolus: N/A - Patch infusor: 36 (30–50)	N/A	- Bolus: 68 (60–79) - Patch infusor: 45 (41–59)	- Bolus: N/A - Patch infusor: 5184 (1922–6488)
Sica et al. 2016 [21]	Prospective Cohort	Netherland	16	N/A	N/A	N/A	N/A	N/A	N/A	N/A	N/A	N/A
Sica et al. 2018 [22]	Prospective Cohort	Netherland	First-in-Man: 10 PK/PD Pivotal: 17	First-in-Man: 8 (80%) PK/PD Pivotal: 15 (88.2%)	First-in-Man: 69.9 ± 8.6 PK/PD Pivotal: 68.0 ± 9.5	First-in-Man: 27.5 ± 4.5 PK/PD Pivotal: 31.0 ± 4.6	N/A	**First-in-Man** - NYHA Class II: 10 (100%) - NYHA Class III: 0 (0%) **PK/PD Pivotal** - NYHA Class II: 13 (76.5%) - NYHA Class III: 4 (23.5%)	N/A	- First-in-Man: 120.0 (102.5–131.2) - PK/PD Pivotal: 105.5 (80.5–143.24)	- First-in-Man: 53.8 (49.5–58.7) - PK/PD Pivotal: 63.4 (41–97)	- First-in-Man: 1130 (732–2,115) - PK/PD Pivotal: 897 (41–2,514)
Afari et al. 2020 [23]	Prospective Cohort	United States	9	3 (33.3%)	75.8 ± 7.6	N/A	N/A	N/A	N/A	N/A	N/A	N/A
Verma et al. 2004 [3]	Prospective Cohort	Canada	12	6 (50%)	36 ± 10	N/A	N/A	N/A	N/A	0.88 ± 0.13	N/A	N/A
Zatarain-Nicolas et al. 2013 [24]	Retrospective Cohort	Spain	24	19 (79%)	75 (10)	N/A	N/A	**Pre** NYHA class III-IV: 22 (93%) **Post** NYHA class III-IV: 12 (49%)	14 (58%)	Pre: 1.57 ± 0.59 Post: 1.52 ± 0.66	N/A	7833

## Quality Assessment

Two authors independently conducted the methodological quality assessment using the Newcastle–Ottawa Assessment Scale [5]. It provides a standardized approach for evaluating the quality of observational studies, focusing on three domains: selection of the research groups, comparability of the groups, and identification of the relevant exposure or outcome. Each study is assigned a score up to a maximum of nine points. None of the included studies exhibited a high risk of bias (Supplementary Table 2). Any discrepancies were resolved by the third author.

## Statistical Analysis

A meta-analysis could not be performed because this systematic review includes a diverse range of data from included studies. Hence, the available evidence was summarized and analyzed qualitatively.

## Results

### Study Selection and Quality Assessment

**Fig. 1:**
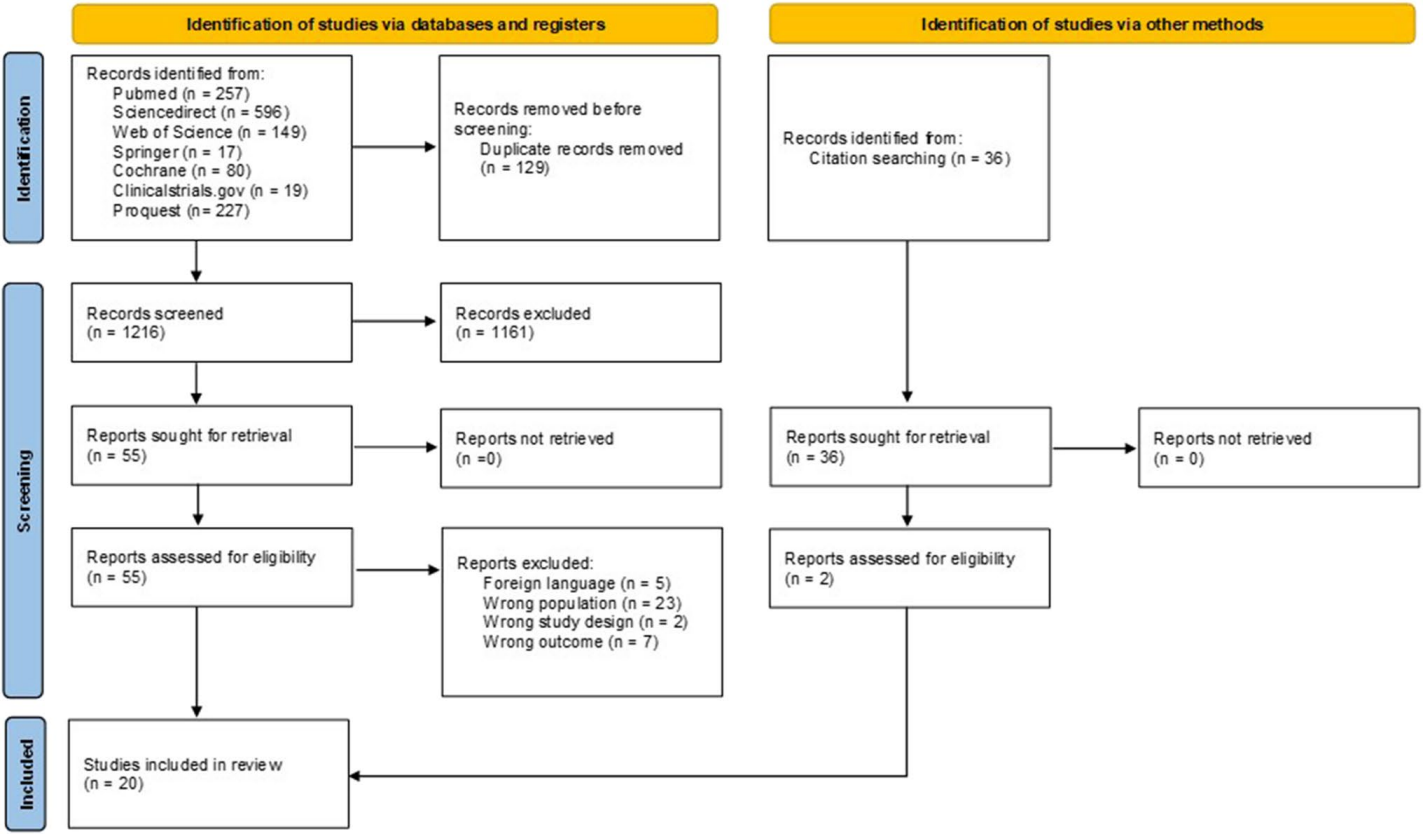
PRISMA flow diagram of the study selection process

Following the initial search, a total of 1216 studies were identified. After resolving 129 duplicates, 55 articles were chosen for review based on their titles and abstracts. The selection procedure for the studies is detailed in the PRISMA flow diagram in Fig. 1, including reasons for exclusion. There was an overlap in subjects between articles by Bensimhon et al. (2023) [6] and Bensimhon et al. (2024) [7]. However, as these articles reported different outcomes, both were included and analyzed separately based on the available outcomes [6, 7]. Additionally, citation searching to encompass gray literature contributed to the inclusion of two more studies [8, 9]. After a thorough full-text assessment, 20 observational studies were included in the systematic review. These eligible studies underwent quality assessment, as detailed in Supplementary Table 2.

## Baseline Characteristic

This review included a total of 687 patients from 20 observational studies. Demographically, most studies were conducted in the United States, United Kingdom, and Spain, followed by Canada and Netherland. The included studies were dominated male with a percentage ranging from 33.3% to 93.8% and aged above 35 years old. In addition, the mean BMI of included patients ranging from 27.5 to 44.4. The characteristics of included studies were summarized in Table 1.

## Characteristics of Subcutaneous Furosemide Utilization

Based on the included studies, indications for patients to receive subcutaneous furosemide are symptomatic HF patients with NYHA class II-IV and volume overload, requiring oral or parenteral diuretic therapy. Most studies reported that the dose of SC furosemide was 80 mg/10 mL for 5 h [6–8, 14, 15, 18, 20–22]. The devices that were used to administer SC furosemide includes SC infusion, syringe pump, and elastomeric pump, with duration of use ranging from 3 to 59 days.

De boer et al. (2018) [14] reported that all patients who received SC furosemide achieved therapeutic plasma levels of Furosemide over 1000 ng/mL within one hour and maintained it for 5 hours with highest blood concentration (Cmax) of 67%-93%. This finding is definitely higher compared to oral Cmax of 11%—40%. Sica et al. (2018) [22] reported that SC furosemide reached therapeutic levels within 30 min and were maintained for the entire drug administration period. The absolute bioavailability of SC furosemide was also similar to IV furosemide, which is 99.65% [22]. Osmanska et al. (2014) [20] also reported similar bioavailability of 112% compared to IV furosemide, resulting in equivalent diuresis and natriuresis. In terms of pharmacodynamic, Verma et al. (2004) [25] documented that mean urine sodium concentration of SC was approximately 4.5 times higher than placebo group. In addition, Sica et al. (2018) [22] reported that total urine volume was similar in both IV and SC furosemide, but urinary sodium excretion was higher in the IV group (367 mmol vs 341 mmol). In addition,. Civera et al. (2022) [17] particularly evaluated certain indicators of congestion at 72 hours and 30 days after SCF utilization. This study reported that there was a sustained increase in uNa + during the first 72 hours of treatment compared to baseline, accompanied by signs of decongestion at both 72 hours and 30 days [17].

## Targeted Outcome following Subcutaneous Furosemide Utilization

Bensimhon et al. (2023) [6] documented that the mean reduction cost of each patient reached up to $16,995 with SC furosemide. The costs were associated with hospitalizations, emergency department (ED) visits and clinic visits within 30 days. In terms of mortality, Austin et al. (2013) [16] mortality was higher in the SC group (SC group n = 10; 91% vs IV group n = 10; 71%). Similar findings also revealed by López‐Vilella et al. (2021) with the utilization of elastomeric pumps. In contrast, Zatarain-Nicolás et al. (2013) reported that there were no treatment-related mortality in their patients.

As the first study that employed SC furosemide, Verma et al. (2004) [3] documented greater urinary output, with the mean total urine volume 3.1 times greater than the placebo and mean urine sodium concentration 4.5 higher than placebo. Civera et al. (2022) [17] additionally found significant improvements in NYHA functional classification, dyspnea VAS scale, pedal edema, weight reduction. Birch et al. (2023) [18], Brown et al. (2019) [19] and Ojeifo et al. (2016) [20] also documented significant weight reduction up to 5 kilograms. Bensimhon et al. (2023) [6] documented that SC group had better impact in alleviating patient symptoms and quality of life, specifically measured with Kansas City Cardiomyopathy Questionnaire – Short Form (KCCQ-12) and NT-proBNP.

## Adverse Events following Subcutaneous Furosemide Utilization

Based on the included studies, there were no major adverse events related to the SC furosemide utilization. Birch et al. (2023) [18] described that most adverse events were related to injection site discomfort. This study also reported practical problems, such as dislodging (n = 5; 3.8%) and leaking (n = 1; 0.8%) during the administration process. Additionally, Zatarain-Nicolás et al. (2013) [21] also reported additional practical problems related to elastomeric pump usage which are disconnection and kinking in 41.7% patients (n = 10) [21], similar to López‐Vilella et al. (2021) [22]. A case of readmission for assistance in syringe driver management was documented by Brown et al. (2019) [19].

Moreover, Bensimhon et al. (2023) [6] reported that there were no occurrences of hypokalemia, hypomagnesemia, hypotension, and worsening renal function (WRF). Conversely, Bensimhon et al. (2024) [7] documented that 9% of patients experienced WRF and hypokalemia particularly in the first 3 days of SC furosemide administration. Moreover, Bensimhon et al. (2024) [7] also revealed that all cases of WRF resolved spontaneously and were not related to SC furosemide doses. Osmanska et al. (2024) [12] also documented an episode of orthostatic hypotension due to early discontinuation of SC infusion.

## Discussion

Most studies found that administering 80 mg of furosemide subcutaneously in HF patients resulted in diuresis and weight loss as effective as IV furosemide [6–8, 14, 15, 18, 20, 22, 23]. Moreover, compared to SC furosemide, higher doses of IV furosemide showed similar urine output volume at 6 h [8]. However, according to Hirsh et al. (2001) [26] the efficacy of the lower dose of SC furosemide may be related to its better bioavailability. This finding is aligned to Verma et al. (2004) [25] that reported 20 mg og SC furosemide produced significantly better diuretic (1430 ± 504 mL vs. 459 ± 279 mL; p < 0.05) and natriuretic (134 ± 31 mEq/L vs. 29 ± 17 mEq/L urine sodium; p < 0.05) effects compared to placebo [25]. In addition, Bensimhon et al. (2024) [7] also reported that SCF utilization was associated with cost reduction related to hospitalizations, emergency department (ED) visits and clinic visits within 30-days [7].

SC furosemide provides a promising alternative that can overcome limitations of IV administration, such as intravenous line insertion by healthcare professionals, incurring maintenance costs and posing inherent risks of infections and thrombosis [27–30]. SC furosemide initially proposed for end-stage HF patients who are unresponsive to high doses of diuretics, with poor venous access and prefer to remain as outpatients, in order to improve patients’ comfort [27, 31, 32]. However, considering the efficacy and safety profile of SC furosemide, it can also be utilized inclusively for ADHF patients requiring parenteral diuretics. Additionally, SC furosemide is more advantageous, as certain patients may encounter temporarily diminished response to oral medications due to compromised intestinal absorptive capacities triggered by excessive fluid accumulation [33, 34]. Thus, SC furosemide may also help to restore oral bioavailability, facilitating transition to oral maintenance therapy [16].

Verma et al. 2004 [3] showed that the mean onset of diuresis with SC administration was 30 min, longer than with the IV route but shorter than the oral route, and similar to the IM route. This suggests that SC furosemide has a relatively rapid onset and moderate duration of pharmacologic action compared to other routes. Regarding efficacy, oral furosemide has demonstrated low bioavailability (72%) with wide variability among individuals [33]. This finding aligns with Gilotra et al. (2018) [8] who reported better bioavailability of SC furosemide compared to oral furosemide [8]. Additionally, Gilotra et al. (2018) [8] also documented that hourly urine output of the SC group was more robust than IV group during the 6 hours observation, demonstrating a consistent absorption rate [22]. This also suggests a more steady level of furosemide achieved through SC administration compared to the initial peak seen with bolus IV administration. Although SC furosemide has lower mean peak plasma concentration, its diuretic effect remains consistent, leading to a greater total urinary output [8]. Civera et al. (2022) [17] also reported that there was a sustained increase of uNa + and signs of decongestion even after 30 days of SC furosemideutilization [17]. It underscores the effectiveness of SC furosemide administration, providing a potent diuretic effect for ADHF patients.

No serious adverse events related to the SC furosemide utilization have been reported. However, López‐Vilella et al. (2021) [16] reported that with a follow-up of 3 years, mortality was higher in the SC group compared to the oral group (80% vs. 71%). This may be explained by the fact that the most frequent cause of death was end-stage HF, and the mortality is not associated with the diuretic treatment itself but is inherent to the poor prognosis associated with HF in these advanced stages of refractory congestion [35]. While the most common adverse event in SC furosemide group is skin irritation, it should be noted that furosemide has an alkaline pH that irritates surrounding skin tissue and allows only low-volume to be injected [36]. Therefore, Alternative strategies to administration of the SC formulation have been the utilization of elastomeric pumps [3, 37]. However, practical limitations may occur due to the catheter placement and continuous infusion [8, 38, 39]. Novel formulations and devices have been developed to deliver furosemide subcutaneously, aiming to overcome these limitations, to improve infusion tolerability and patient comfort. Osmanska et al. (2014) [20] particularly utilized a novel abdominal patch infusor device that was adapted from an insulin pump with a proven history of user-friendly operation. A formulation of isotonic furosemide solution with a pH range of 7.0 to 7.8 has also been developed for subcutaneous administration [38]. Moreover, a preprogrammed delivery system for subcutaneous furosemide such as Furoscix was developed to address practical limitations associated with administering furosemide subcutaneously. Furoscix infusor consists of a reusable activator and a cost-efficient single-use cartridge with a micro-piston pump. Over a span of 5 hours, a total of 80 mg of furosemide is delivered subcutaneously, with an initial dose of 30 mg in the first hour followed by a continuous infusion of 12.5 mg/hour for the subsequent 4 hours [6–8, 13].

## Recommendation in Daily Practices and Incorporation to Latest Guidelines

Although long-term mortality rates necessitate further investigation, included studies consistently highlighted its effectiveness in alleviating symptoms among symptomatic HF patients with volume overload, especially when oral diuretic treatments prove insufficient against worsening congestion. The administration of 80 mg of SC furosemide over a 5 hours period has demonstrated comparable diuretic and weight loss outcomes to IV furosemide, rapidly achieving and sustaining therapeutic plasma levels. Moreover, SC furosemide also has comparable bioavailability with IV administration, ensuring similar diuretic and natriuretic effects. Despite practical limitations and minor adverse events, SC furosemide administration has generally been well-tolerated by both patients and their caregivers. As advancements in formulation and infusion devices continue, the integration of SC furosemide into routine clinical practices, and possibly into future guidelines, can be considered for optimizing the management of HF.

## Study Strengths and Limitations

This study has several notable strengths and limitations. There is a lack of existing studies that provide comprehensive data regarding the efficacy and safety of SC furosemide. Additionally, included studies also had relatively short follow-up periods. Despite systematic attempts to gather and analyze pertinent data, meta-analysis could not be performed. Despite these constraints, this study stands as the first comprehensive review that evaluated the efficacy and safety of SC furosemide. The screening process was meticulously conducted by two independent reviewers, and the drafting of the manuscript closely adhered to PRISMA protocols. Finally, these constraints underscores the necessity for future studies with larger populations, to accumulate more robust evidence for establishing precise clinical guidelines.

## Conclusions

In conclusion, SC furosemide offers a promising alternative for managing ADHF, especially in NYHA class II-IV patients with volume overload. Its utilization results in significant cost reductions, symptom relief, and improved quality of life. Although further investigation into mortality rates is needed, SC furosemide demonstrates efficacy comparable to IV furosemide in diuresis and weight loss, with similar bioavailability and natriuretic effects. Adverse events are generally minor, predominantly comprising skin irritation. Strategies like developing isotonic alkaline solutions and preprogrammed infusion devices like Furoscix aim to address these challenges. Integration of SC furosemide into routine practices, and future guidelines, could optimize HF management.

## Key References

- Bensimhon D, Weintraub WS, Peacock WF, Alexy T, McLean D, Haas D, et al. Reduced heart failure-related healthcare costs with Furoscix versus in-hospital intravenous diuresis in heart failure patients: the FREEDOM-HF study. Future Cardiol. 2023;19(8):385–96.
  - This study highlights significant reductions in healthcare costs associated with HF management, demonstrating the potential cost-effectiveness of SCF over traditional in-hospital intravenous diuresis.
- Civera J, de la Espriella R, Heredia R, Miñana G, Santas E, Conesa A, et al. Efficacy and Safety of Subcutaneous Infusion of Non-formulated Furosemide in Patients with Worsening Heart Failure: a Real-World Study. J Cardiovasc Transl Res. 2022;15(3):644–52.
  - This study showed the efficacy of SCF for decongestion managements in ADHF patients based on clinical and laboratory parameters, without any significant adverse effect.
- Osmanska J, Brooksbank K, Docherty KF, Robertson S, Wetherall K, McConnachie A, et al. A novel, small-volume subcutaneous furosemide formulation delivered by an abdominal patch infusor device in patients with heart failure: results of two phase I studies. Eur Heart J Cardiovasc Pharmacother. 2024;10(1):35–44.
  - This study emphasized the innovation of SC furosemide delivery methods and revealed the comparable  pharmacokinetics, pharmacokinetics and safety profile of SC furosemide to IV furosemide.

## Supplementary Information

Below is the link to the electronic supplementary material.

Supplementary file1 (DOCX 248 KB)

## Data Availability

No datasets were generated or analysed during the current study.
