# Supplementary material for: The Role of Subcutaneous Furosemide in Heart Failure Management: A Systematic Review
Source: Curr Cardiol Rep. 2024 Oct 1;26(11):1285–96. doi: 10.1007/s11886-024-02124-4 (PMC11538160; doi:10.1007/s11886-024-02124-4)
Supplement: Supplementary file 1 — Supplementary file1 (DOCX 248 KB) [file 11886_2024_2124_MOESM1_ESM.docx]

**Title: S**ubcutaneous Furosemide Utilization: Is it Truly More Beneficial?

A Comprehensive Systematic Review

**Supplementary Files**

**Supplementary Figure 1.** PRISMA checklist

**Supplementary Table 2.** Risk of Bias Assessment of Included Observational Studies according to the Newcastle-Ottawa Assessment Scales

**Supplementary Table 3** Comorbidities and Additional Details regarding Included Patients

**Supplementary Tabel 4** Administration of SC Furosemide in Included Studies and Its Outcome

**Supplementary Figure 1.** PRISMA checklist


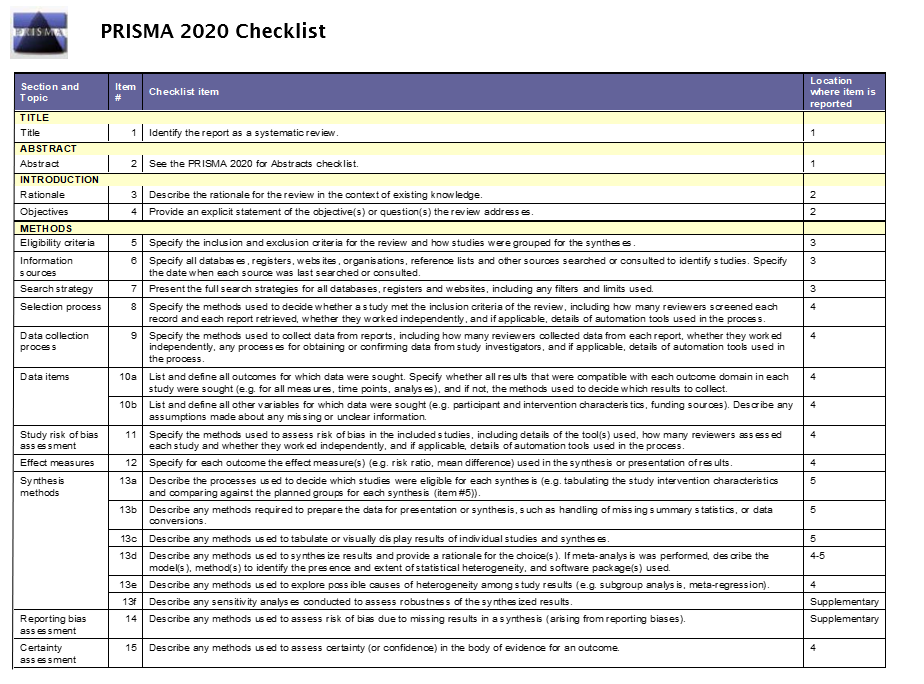


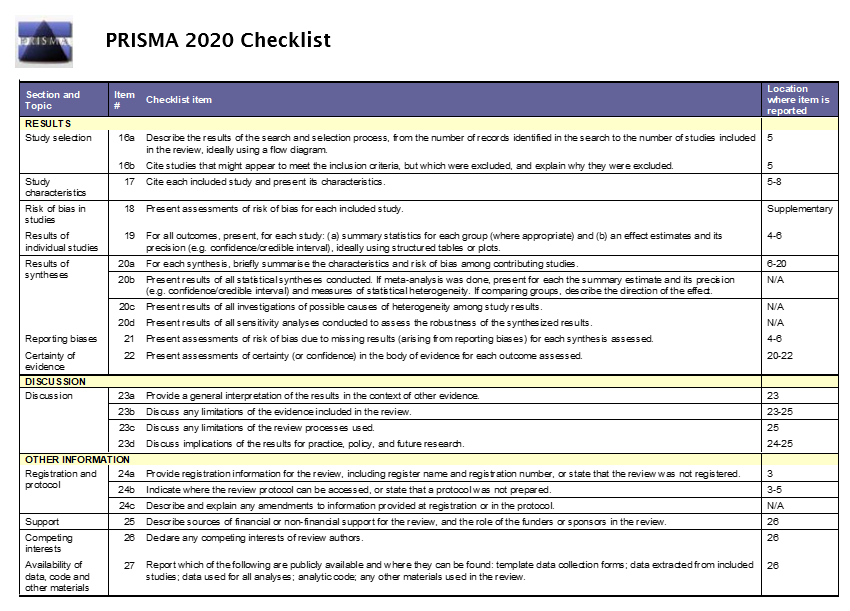


**Supplementary Table 2.** Risk of Bias Assessment of Included Observational Studies according to the Newcastle-Ottawa Assessment Scales

| **Author, Year** | **Selection** | | | | **Comparability** | **Outcome** | | | **Total** | **Category** |
| --- | --- | --- | --- | --- | --- | --- | --- | --- | --- | --- |
|  | Q1 | Q2 | Q3 | Q4 | Q1 | Q1 | Q2 | Q3 |  |  |
| Austin et al., 2013 | 1 | 1 | 1 | 1 | 2 | 1 | 1 | 1 | 9 | Good Quality |
| Bensimhon  et al., 2023 | 1 | 1 | 1 | 0 | 1 | 1 | 1 | 0 | 6 | Good Quality |
| Bensimhon  et al., 2024 | 1 | 1 | 1 | 1 | 2 | 1 | 1 | 1 | 9 | Good Quality |
| Birch et al., 2023 | 1 | 1 | 1 | 0 | 1 | 1 | 1 | 1 | 7 | Good Quality |
| Brown et al., 2019 | 1 | 1 | 1 | 1 | 2 | 0 | 1 | 1 | 8 | Good Quality |
| Civera et al., 2022 | 1 | 1 | 1 | 1 | 2 | 1 | 1 | 1 | 9 | Good Quality |
| De boer et al., 2016 | 1 | 1 | 1 | 0 | 2 | 1 | 0 | 1 | 7 | Good Quality |
| Felker et al., 2019 | 1 | 1 | 1 | 1 | 1 | 1 | 0 | 1 | 7 | Good Quality |
| Galindo-ocana et al., 2012 | 1 | 1 | 1 | 0 | 1 | 1 | 1 | 1 | 7 | Good Quality |
| Gilotra et al., 2018 | 1 | 1 | 1 | 1 | 2 | 1 | 1 | 1 | 9 | Good Quality |
| López‐Vilella et al., 2021 | 1 | 1 | 1 | 1 | 2 | 1 | 1 | 1 | 9 | Good Quality |
| Lozano Bahamonde  et al., 2018 | 1 | 1 | 1 | 1 | 1 | 1 | 1 | 1 | 8 | Good Quality |
| Mohr et al., 2018 | 1 | 1 | 1 | 1 | 2 | 0 | 0 | 1 | 7 | Good Quality |
| Ojeifo et al., 2016 | 1 | 1 | 1 | 1 | 2 | 0 | 1 | 1 | 8 | Good Quality |
| Osmanska  et al., 2024 | 1 | 1 | 1 | 1 | 2 | 1 | 1 | 1 | 9 | Good Quality |
| Sica et al., 2016 | 1 | 1 | 1 | 1 | 2 | 1 | 1 | 0 | 8 | Good Quality |
| Sica et al., 2018 | 1 | 1 | 1 | 1 | 2 | 1 | 1 | 1 | 9 | Good Quality |
| Afari et al., 2020 | 1 | 1 | 1 | 1 | 1 | 1 | 1 | 0 | 7 | Good Quality |
| Verma et al., 2004 | 1 | 1 | 1 | 1 | 2 | 0 | 1 | 1 | 8 | Good Quality |
| Zatarain-Nicolas et al., 2013 | 1 | 1 | 1 | 1 | 2 | 1 | 1 | 1 | 9 | Good Quality |

**Supplementary Table 3** Comorbidities and Additional Details regarding Included Patients

| **Author, Year** | **Comorbidities** | | | | | **Diuretic** | | | **Medication** | | | |
| --- | --- | --- | --- | --- | --- | --- | --- | --- | --- | --- | --- | --- |
|  | **DM** | **CKD** | **HT** | **CAD** | **COPD** | **Acetazolamide** | **Spironolactone (MRA)** | **Tolvaptan** | **BB** | **ACEi/ARB** | **ARNi** | **SGLT2i** |
| Austin et al., 2013 | N/A | N/A | N/A | N/A | N/A | N/A | N/A | N/A | N/A | N/A | N/A | N/A |
| Bensimhon  et al., 2023 | **Furoscix**: 12 (50.0%)  **Comparator**: 50 (75.8%) | **Furoscix**  CKD Stage 2: 1 (4.2%)  CKD Stage 3: 6 (25.0%)  **Comparator**  CKD Stage 2: 4 (6.1%)  CKD Stage 3:17 (25.8%) | **Furoscix**: 22 (91.7%)  **Comparator**: 64 (97.0%) | **Furoscix**: 6 (25.0%)  **Comparator**:17 (25.8%) | **Furoscix**: 6 (25.0%)  **Comparator**: 27 (40.9%) | N/A | Furoscix: 10 (41.7%)  Comparator: 12 (18.2%) | N/A | Furoscix: 15 (62.5%)  Comparator: 33 (50.0%) | Furoscix: 11 (45.8%)  Comparator: 52 (78.8%) | | N/A |
| Bensimhon  et al., 2024 | N/A | CKD St.2: 22 (38%)  CKD St.3A: 13 (24%)  CKD St.3B: 13 (24%) | N/A | N/A | N/A | N/A | N/A | N/A | N/A | N/A | N/A | N/A |
| Birch et al., 2023 | N/A | N/A | N/A | N/A | N/A | N/A | N/A | N/A | N/A | N/A | N/A | N/A |
| Brown et al., 2019 | N/A | N/A | N/A | N/A | N/A | N/A | N/A | N/A | N/A | N/A | N/A | N/A |
| Civera et al., 2022 | N/A | N/A | 48 (87.3%) | N/A | 4 (7.3%) | 1 (1.8%) | 44 (80%) | N/A | 51 (92.7%) | 25 (60%) | 15 (27.3%) | 13 (23.6%) |
| De boer et al., 2016 | N/A | N/A | N/A | N/A | N/A | N/A | N/A | N/A | N/A | N/A | N/A | N/A |
| Felker et al., 2019 | N/A | N/A | N/A | N/A | N/A | N/A | N/A | N/A | N/A | N/A | N/A | N/A |
| Galindo-ocana et al., 2012 | N/A | N/A | N/A | N/A | N/A | N/A | N/A | N/A | N/A | N/A | N/A | N/A |
| Gilotra et al., 2018 | - IV: 12 (63%)  - SQ: 7 (33%) | - IV: 9 (47%)  - SQ: 11 (52%) | - IV: 12 (63%)  - SQ: 18 (86%) | - IV: 8 (42%)  - SQ: 6 (29%) | - IV: 4 (21%)  - SQ: 4 (19%) | N/A | N/A | N/A | - IV: 15 (79%)  - SQ:16 (76%) | - IV: 10 (53%)  - SQ:13 (62%) | N/A | N/A |
| López‐Vilella et al., 2021 | - SC: 3 (30%)  - Oral: 8 (47.1%) | N/A | - SC: 4 (40%)  - Oral: 14 (82.4%) | N/A | N/A | - SC: 0 (0%)  - Oral: 2 (11.8%) | - SC: 6 (60%)  - Oral: 11 (64.7%) | - SC: 6 (60%)  - Oral: 5 (29.4%) | - SC: 5 (50%)  - Oral: 8 (47%) | - SC: 0 (0%)  - Oral: 2 (12%) | - SC: 1 (10%)  - Oral: 6 (35%) | - SC: 0 (00%)  - Oral: 6 (35.3%) |
| Lozano Bahamonde  et al., 2018 | 6 (50%) | N/A | 11 (91.7%) | N/A | N/A | N/A | N/A | N/A | N/A | N/A | N/A | N/A |
| Mohr et al., 2018 | N/A | N/A | 16 (100%) | N/A | N/A | N/A | N/A | N/A | N/A | N/A | N/A | N/A |
| Ojeifo et al., 2016 | N/A | N/A | N/A | N/A | N/A | N/A | N/A | N/A | N/A | N/A | N/A | N/A |
| Osmanska  et al., 2024 | - Bolus: 6 (33%)  - Patch infusor: 7 (35%) | N/A | - Bolus: 18 (100%)  - Patch infsuor: 11 (55%) | N/A | N/A | N/A | - SQIN-Furosemide PK/PD  study: 4 (22%)  - SQIN-Furosemide/abdominal  device trial: 7 (35%) | N/A | - Bolus: 13 (72%)  - Patch infusor: 12 (60%) | - Bolus: 16 (89%)  - Patch infusor: 13 (65%) | N/A | - Bolus: 0 (0%)  - Patch infusor: 4 (20%) |
| Sica et al., 2016 | N/A | N/A | N/A | N/A | N/A | N/A | N/A | N/A | N/A | N/A | N/A | N/A |
| Sica et al., 2018 | First-in-Man: 1 (10%)  PK/PD Pivotal: 6 (35.3%) | N/A | N/A | N/A | N/A | N/A | N/A | N/A | N/A | N/A | N/A | N/A |
| Afari et al., 2020 | N/A | N/A | N/A | N/A | N/A | N/A | N/A | N/A | N/A | N/A | N/A | N/A |
| Verma et al., 2004 | N/A | N/A | N/A | N/A | N/A | N/A | N/A | N/A | N/A | N/A | N/A | N/A |
| Zatarain-Nicolas et al., 2013 | 5 (21%) | 9 (38%) | 12 (50%) | N/A | N/A | N/A | N/A | N/A | 18 (73%) | 20 (81%) | N/A | 6 (27%) |

**Supplementary Table 4. Administration of SC Furosemide in Included Studies and Its Outcome**

| **Author, Year** | **SCF** | | | | | **Decongestion marker** | **Potassium** | **Sodium** | **Urea** | **Adverse Events** |
| --- | --- | --- | --- | --- | --- | --- | --- | --- | --- | --- |
|  | **Indication** | **Dose** | **Frequency** | **Duration of use** | **Method of use** |  |  |  |  |  |
| Austin et al., 2013 | Patients classified as NYHA IIIb/IV with sign and symptoms of fluid overload (> 3 kg from baseline) and  medically unfit for IV treatment (having hypotension, poor  venous access or under the auspices of palliative care) | slow bolus at 40mg daily, increasing up to a maximum of 80mg | 2x/day | 59 days  (73% of patients ≤.7 days) | Grasby MS26 syringe pump with a flexible silicon catheter (soft set) | Edema | N/A | N/A | N/A | - No major adverse events following furosemide infusion |
| Bensimhon  et al., 2023 | Patients were 18–80 years old, had chronic HF (NYHA class II-III), were on background oral diuretic therapy (40–160 mg furosemide equivalent daily), and presented to the ED with signs and symptoms directly related to worsening congestion | 80 mg/10 ml for 5 hours (daily furosemide equivalent dose 139.1 ± 98.1) | 1-2x/day up to 7 doses | ≤ 7 days | Subcutaneous infusion | N/A | N/A | N/A | N/A | - The most frequently reported adverse events consisted of infusion site bruising (29.2%), infusion site pain  (29.2%) and dizziness (12.5%).  - No hypokalemia, hypomagnesemia, hypotension or worsening renal function  related to study drug use were reported. |
| Bensimhon  et al., 2024 | Hemodynamically stable patients with NYHA Class II-IV  chronic HF with worsening signs and symptoms of congestion requiring  augmented diuresis | 80 mg/10 mL for 5 hours | - AT HOME-HF: 4 (1-9)  -FRREDOM-HF: 6 (2-10) | 30 days (83% ≤ 7 days; 61% ≤ 3 days) | Subcutaneous infusion | N/A | Increased by a mean of 0.01 ± 0.6 mg/dL. | N/A | N/A | - 5 (9%) patients experienced WRF (2 patients after 1 dose, 2 patients after 3  doses and 1 patient after 4 doses)  - 5 patients (9%) developed hypokalemia during the first 3 days |
| Birch et al., 2023 | Patients from a regional heart failure-palliative care multi-disciplinary service  (heart failure nurses, palliative physician, cardiologists) who had optimised heart failure management; required parenteral diuretic; had a preferred place of care as home, hospice or care home; had sufficient community  support (informal and clinical). | 124.8 ± 49.6  (40 to 250mg)  (Starting dose was estimated based on previous 24 hours oral dose) | 130 | 10 (1-49) days | Subcutaneous continuous infusion of furosemide (CSCI-furosemide) using syringe driver over 24 hours | self-reported breathlessness, weight, JVP, edema, crackles | N/A | N/A | N/A | Adverse events occurred in 31 (23.8%) episodes;  - Skin infection: 4 (3.1%)  - Self limiting skin infection: 18 (13.8%)  - Practical problem: 6 (4.6%)  - |
| Brown et al., 2019 | End-stage heart failure patients with e a comprehensive emergency healthcare plan (EHCP) involving the heart failure team and delivery of the service is facilitated by community heart failure and district nurses in patients’ homes, requiring parenteral diuretics to relieve symptoms and avoid hospital admission | N/A | 19 | 10 (5–19) days. | N/A | weight | N/A | N/A | N/A | N/A |
| Civera et al., 2022 | Worsening HF with at least one of the following inclusion criteria: (1) structural or functional cardiac abnormality; (2) NT-proBNP >1000 pg/mL; (3) history of symptomatic chronic HF on active treatment with oral loop diuretics | 100 mg daily | N/A | 72 hours | Infusion pump with elastomeric balloon | Pedal edema, pleural effusion, jugular engorgement | 4.2 ± 0.5 | 139.6 ± 4.1 | 74 (51 – 119) | N/A |
| De boer et al., 2016 | Stable chronic HF | 80 mg/mL for 5 hours | N/A | 5 hours | SC administration with a syringe pump over for 5 hours using a biphasic pattern (30mg over 60 min followed by  12.5mg/hour for 4 hours.). | N/A | N/A | N/A | N/A | N/A |
| Felker et al., 2019 | Patients hospitalized for HF with congestion | 80mg sc over 5 hours given QD or BID based on need | - IP: 2.9  - OP: 6.9 | - IP: 48 hours  - OP: 7 days | N/A | Urine output, weights, symptoms, | N/A | N/A | N/A | - There were 18 AE’s (mostly mild and related to injection site discomfort). There was 1 SAE that was unrelated to study treatment. |
| Galindo-ocana et al., 2012 | All DAHF patients that were older than 18 years,  (NYHA) class III or IV symptoms, treated at home by a  Southern Spanish university hospital at-home unit. | Daily: 160 (99–250)  Total: 720 (480–1125) | 63 | N/A | N/A | N/A | N/A | N/A | N/A | - There were 5 adverse events (3 haematomas, 1 severe local skin infection, with favourable outcome  under oral antibiotics; and 1 drip removal) |
| Gilotra et al., 2018 | Adult outpatients who presented with a history of HF treatment at least 3 months or HF hospitalization within 60 days and who were identified by providers as requiring IV diuresis for worsening HF were recruited then randomly assigned | 80 mg of scFurosemide administered over 5 h (30 mg in the first hour, followed by 12.5 mg/h for 4 h) | N/A | 30 days | Infusion pump system (Perfusor space infusion pump, B. Braun Medical, Bethlehem, Pennsylvania) | S3 heart sound, Jugular venous distension, rales/ diminshed breath sounds, lower extremity edema, dyspnea on exertion, orthopnea/ paroxysmal nocturnal dyspnea, fatigue | N/A | - IV: 139 (137–142)  - SQ: 139 (137–140) | N/A | - There was no worsening renal function, ototoxicity, or skin irritation with either formulation |
| López‐Vilella et al., 2021 | Patients with diuretic resistance who were not candidates for peritoneal dialysis, outpatient ultrafltration, regular doses of levosimendan, ventricular assistance, or heart transplantation were recruited. Mode of treatment was chosen based on the proximity and accessibility of the patient to the center for control and removal of the elastomeric pump. | 100 mg daily | N/A | 5 days | Elastomeric pump | N/A | - SC: 3.9 ± 0.6  - Oral: 4.3 ± 0.6 | - SC: 138 ± 5  - Oral: 141 ± 4 | - SC: 106 ± 59  - Oral: 121 ± 70 | - 1 patient treated with a subcutaneous pump required  replacement of the device with a kink in the tubing that blocked the drug’s fow  - 1 patient experienced skin irritation requiring a change  of the furosemide infusion area |
| Lozano Bahamonde  et al., 2018 | Patients with decompensated HF without  response to oral diuretics, frequent hospital admissions or repetitive  use of intravenous treatment at home. | 135.0 ± 30.90 | N/A | N/A | Elastomeric pump | N/A | N/A | N/A | N/A | - The treatment was withdrawn in 1 patient due to local complications.  - 2 infectious complications were found and managed with antibiotic treatment  - Despite the high prevalence of chronic renal failure, no significant renal impairment or  hydroelectrolytic derangements were detected. |
| Mohr et al., 2018 | Patients with HF were recruited for randomized,  open label, 2-way crossover study | 80 mg was administered over 5 hours in a biphasic regimen (30 mg 1st hour followed by 12.5 mg/hr for the subsequent 4 hours) | N/A | N/A | N/A | N/A | N/A | N/A | N/A | N/A |
| Ojeifo et al., 2016 | Chronic HF patients who present with clinical evidence of volume overload are randomized to receive  either a bolus of IV furosemide or a sc infusion of furosemide in a non-blinded fashion | 30 mg over the first hour followed by 12.5 mg per hour over the subsequent 4 hours for a total of 80 mg | N/A | 30 days | B Braun Perfusor Space Infusion System | Urine output,  weight loss,  symptoms | N/A | N/A | N/A | - Transient hypokalemia and significant worsening of renal function have not occurred in either the IV or SC furosemide cohorts. |
| Osmanska  et al., 2024 | Patients had chronic HF, NYHA class II or III, treated with oral furosemide at a dose of ≥40 mg per day and estimated glomerular filtration rate (eGFR) ≥45 mL/min per 1.73 m2 were recruited in an open-label, single-dose, randomized, active-comparator, crossover single-centre study | 80 mg | N/A | 7 days | - Bolus: Medfusion 3500 (v6) precision infusion pump  - Patch infusor: abdominal patch infusor pump | Urine output | - Bolus: 4.2 [3.8–4.7]  - Patch infusor: 4.1 [3.9–4.3] | - Bolus: 140 [139–143]  - Patch infusor: 138 [137–142] | N/A | - Bolus: 2 AEs were attributed to study treatment: (1) maxi-  mum pain at the infusion site reported as 6 out of 10 (subsequently improved to 2 out of 10) and (2) an episode of orthostatic hypotension resulting in early discontinuation of SC infusion (10 min before the planned end of infusion)  -Patch infusor: there were no treatment-related adverse events. |
| Sica et al., 2016 | Randomized open-label crossover study in patients with stable HF | 80 mg (8 mg/mL in 10 mL) with over for 5 hours using a biphasic pattern (30 mg over 60 min followed by 12.5 mg/hour for 4 hours) | N/A | N/A | N/A | N/A | N/A | N/A | N/A | N/A |
| Sica et al., 2018 | HF patients with chronic fluid overload were recruited in single-center, randomized, open-label, cross-over exploratory study | 80 mg (8 mg/ml in 10 ml). 30 mg over the first 60 min followed by 12.5 mg/h for 4 h) | N/A | 14 days | External infusion pump | N/A | - First-in-Man: 4.5 (4.3–4.7)  - PK/PD Pivotal: 4.7 (4.0–5.6) | - First-in-Man: 140.0 (138.2–141.5)  - PK/PD Pivotal: 142.2 (135–147) | N/A | - None of the subjects reported pain at the injection site during or after SC administration.  - Only 1 patient reported transient “very slight erythema” during SC furosemide administration.  - 8 subjects were found to have minimal erythema after completion of the infusion and removal of the infusion set and adhesive  - A total of 6 subjects were found to have minimal swelling during or following SC administration |
| Afari et al., 2020 | Symptomatic NYHA Class II-III HF patients were enrolled in a prospective, open-label study | A total dose of 80 mg SC furosemide was infused at 30 mg over the first hour and then at 12.5 mg per hour over the subsequent 4 hours | N/A | 7 days | sc2WearTM Furosemide Infusor pump | N/A | N/A | N/A | N/A | N/A |
| Verma et al., 2004 | Healthy volunteers were recruited in a single-center, double-blind, placebo-controlled, randomized, cross-  over pilot study. The inclusion criteria were no known medical conditions, currently using no medications, no known allergies to furosemide or sulfonamides, not pregnant, and normal kidney function as determined by measurement of blood urea nitrogen (BUN) and serum creatinine | Furosemide 20 mg (2 mL) | N/A | 5 days | Subcutaneous injection | Urine output | N/A | N/A | N/A | N/A |
| Zatarain-Nicolas et al., 2013 | Decompensated HF patients in the Heart Failure Unit | 96 mL at 1 mL/h or 240 mL at 2 mL/h | N/A | 4 or 5 days | Elastomeric pump that were connected to a catheter (Abbocath 20-22GW) implanted subcutaneously in chest or abdominal tissue | N/A | N/A | N/A | N/A | Local complications  - Infection/abscess 7 (29.2%)  - Irritation 12 (50.0%)  - Disconnection/kinking 10 (41.7%) |
